# Ropporin-1 and 1B Are Widely Expressed in Human Melanoma and Evoke Strong Humoral Immune Responses

**DOI:** 10.3390/cancers13081805

**Published:** 2021-04-09

**Authors:** Jessica Da Gama Duarte, Katherine Woods, Luke T. Quigley, Cyril Deceneux, Candani Tutuka, Tom Witkowski, Simone Ostrouska, Chris Hudson, Simon Chang-Hao Tsao, Anupama Pasam, Alexander Dobrovic, Jonathan M. Blackburn, Jonathan Cebon, Andreas Behren

**Affiliations:** 1 Olivia Newton-John Cancer Research Institute, Heidelberg, VIC 3084, Australia; jessica.duarte@onjcri.org.au (J.D.G.D.); katherine@nrlquality.org.au (K.W.); luke.quigley@onjcri.org.au (L.T.Q.); cyril.deceneux@rmit.edu.au (C.D.); dtutuka@akoyabio.com (C.T.); tom.witkowski@onjcri.org.au (T.W.); simone.ostrouska@onjcri.org.au (S.O.); chudson@phcn.vic.gov.au (C.H.); simon.tsao@svha.org.au (S.C.-H.T.); anu.pasam@petermac.org (A.P.); alexander.dobrovic@unimelb.edu.au (A.D.); j.cebon@onjcri.org.au (J.C.); 2 School of Cancer Medicine, La Trobe University, Bundoora, VIC 3086, Australia; 3 Department of Clinical Pathology, Melbourne Medical School, University of Melbourne, Parkville, VIC 3010, Australia; 4 Department of Integrative Biomedical Sciences, Faculty of Health Sciences, University of Cape Town, Cape Town 7925, South Africa; jonathan.blackburn@uct.ac.za; 5 Institute for Infectious Disease and Molecular Medicine, University of Cape Town, Cape Town 7925, South Africa; 6 Medical Oncology Unit, Austin Health, Heidelberg, VIC 3084, Australia; 7 Department of Medicine—Austin, Melbourne Medical School, University of Melbourne, Parkville, VIC 3010, Australia

**Keywords:** melanoma, tumour antigens, Ropporin-1, ROPN1, Ropporin-1B, ROPN1B

## Abstract

**Simple Summary:**

Despite the unprecedented clinical benefit of immunotherapy in melanoma, some patients still do not respond to treatment, arguing the need for novel therapeutic targets. The aim of this study was to investigate the therapeutic potential of two understudied proteins, Ropporin-1 (ROPN1) and 1B (ROPN1B). We confirmed that these proteins are widely expressed in melanoma patients using gene data derived from public datasets, and protein data derived from 61 patient tumours. Moreover, these proteins were able to evoke strong immune responses in 104 melanoma patients. These findings therefore suggest that ROPN1 and ROPN1B may be valuable targets for immunotherapy, alone or in combination with existing treatments.

**Abstract:**

Antibodies that block immune regulatory checkpoints (programmed cell death 1, PD-1 and cytotoxic T-lymphocyte-associated antigen 4, CTLA-4) to mobilise immunity have shown unprecedented clinical efficacy against cancer, demonstrating the importance of antigen-specific tumour recognition. Despite this, many patients still fail to benefit from these treatments and additional approaches are being sought. These include mechanisms that boost antigen-specific immunity either by vaccination or adoptive transfer of effector cells. Other than neoantigens, epigenetically regulated and shared antigens such as NY-ESO-1 are attractive targets; however, tissue expression is often heterogeneous and weak. Therefore, peptide-specific therapies combining multiple antigens rationally selected to give additive anti-cancer benefits are necessary to achieve optimal outcomes. Here, we show that Ropporin-1 (ROPN1) and 1B (ROPN1B), cancer restricted antigens, are highly expressed and immunogenic, inducing humoral immunity in patients with advanced metastatic melanoma. By multispectral immunohistochemistry, 88.5% of melanoma patients tested (*n* = 54/61) showed ROPN1B expression in at least 1 of 2/3 tumour cores in tissue microarrays. Antibody responses against ROPN1A and ROPN1B were detected in 71.2% of melanoma patients tested (*n* = 74/104), with increased reactivity seen with more advanced disease stages. Thus, ROPN1A and ROPN1B may indeed be viable targets for cancer immunotherapy, alone or in combination with other cancer antigens, and could be combined with additional therapies such as immune checkpoint blockade.

## 1. Introduction

Immune checkpoint blockade (ICB) targeting programmed cell death 1 (PD-1) and cytotoxic T-lymphocyte-associated antigen 4 (CTLA-4) has revolutionised the treatment of melanoma, with clinical benefit seen in up to 70% of patients when compared to either PD-1 (54%) or CTLA-4 blockade (41%) alone [1]. This is mediated by the induction or reactivation of antigen-specific effector T lymphocytes. There is considerable evidence that the peptide products of mutated genes [2] or aberrant post-translational changes are important immune targets in cancer [3]. These neoantigens have been correlated with patient responses to ICB. Additionally, a study in melanoma demonstrated immunogenicity of personalised neoantigen vaccines, designed to selectively target the mutated antigens of each patient [4]. However, next-generation sequencing and algorithms for human leukocyte antigen (HLA) binding indicate that as many as two thirds of human cancers do not generate mutational neoantigens at sufficiently high frequencies to ensure immune recognition [5,6]. Clearly, approaches that extend options for immunotherapy need to also account for tumours with low numbers or absent mutated antigens. Indeed, there is abundant evidence in pre-clinical models and in human clinical trials, indicating that ICB can be highly effective if combined with vaccines or adoptive cell transfer (ACT) of effector T lymphocytes, even in tumour models with few mutations [7,8,9]. Thus, combinations with antigen-specific therapeutic approaches are amendable for increasing the scope of ICB as long as appropriate antigens with optimal characteristics can be identified.

For practical purposes, vaccines that utilise shared antigens are attractive since this avoids the complexity and expense of generating customised products for individual patients. For such vaccines, the epigenetically-regulated cancer-testis antigens (CTAgs) can be considered as immune-neoantigens, since their expression is newly acquired as part of the malignant phenotype. A variety of CTAgs have been widely studied for their use as vaccine antigens or for ACT [10,11,12,13]. Arguably, the prototype CTAg is NY-ESO-1 (*CTAG1A* or identical gene copy *CTAG1B*), which induces spontaneous cellular and humoral immune responses in melanoma and other cancers [14,15,16]. However, while CTAgs have the distinct advantage of being tumour-specific, expression is often heterogeneous, patchy or weak [17]. To overcome this, cancer vaccines have been designed using several immunogenic epitopes from different CTAgs in combination [18,19,20]. To date, success with these treatment types has been limited. However, peptide vaccines are currently being reconsidered as combination treatments with ICB and ACT to boost immunity and broaden the cohort of responders [21,22,23]. Thus, identification of novel tumour-specific antigens continues to be of importance in the development of effective anti-cancer therapies. The ideal antigen would be one that is immunogenic and expressed in a large percentage of cancer patients, while limited in any normal tissue expression. Here, we describe and characterise ropporin-1 (ROPN1) and 1B (ROPN1B, 96% sequence homology with ROPN1) as antigens with the aforementioned properties. Based on studies of tissue distribution, CTAgs have been broadly classified as testis-restricted, testis/brain-restricted, and testis-selective, with ROPN1 being classified as the latter [24]. Despite this, the testis is an immune-privileged site that is protected from systemic immune attack, and hence ROPN1 or ROPN1B-specific therapeutic approaches should not result in testicular toxicity [25]. Hence, although poorly studied, we propose they represent promising targets for further clinical development.

Ropporin was firstly described in 1999 as a testis-restricted, rhophilin-binding protein by Fujita et al., using a yeast two hybrid screen on a mouse testis cDNA library [26]. Human ropporin has been shown to directly interact with and bind to A-kinase anchoring proteins (AKAPs) [27], predominantly AKAP110. While the exact function of ropporin remains elusive, its localization in sperm cells and expression data from patients with asthenozoospermia suggested a potential role in sperm motility [28]. The expression pattern of human ropporin is tissue-restricted, with the majority of expression detected in testis and foetal liver, as well as a variety of hematologic malignancies [29] and breast cancers [30].

A recent study using triple negative breast cancer cells suggests that ROPN1 activates RhoA signalling via rhophilin-1 (RHPN1), promoting cell migration, invasion and metastatic potential [30]. It was shown to be overexpressed in triple negative breast cancer cell lines and tissue, with high levels predictive of a poor prognosis [30]. Ropporin was identified as a potential target for immunotherapy in multiple myeloma, where its expression was detected in 44% of cases and its immunogenic potential was confirmed by the presence of antibodies and cytotoxic lymphocytes [31]. We therefore hypothesised that this may also be the case in melanoma and aimed to investigate the expression and immunogenicity of ROPN1 and ROPN1B. Here, we found that both ROPN1A and ROPN1B have favourable features as tumour antigens based on distribution and immunogenicity.

## 2. Results

### 2.1. Ropporin-1 (ROPN1) and Ropporin-1B (ROPN1B) Genes Are Expressed in Melanoma Samples and Correlated with Melanoma Differentiation Antigens

**Figure 1:**
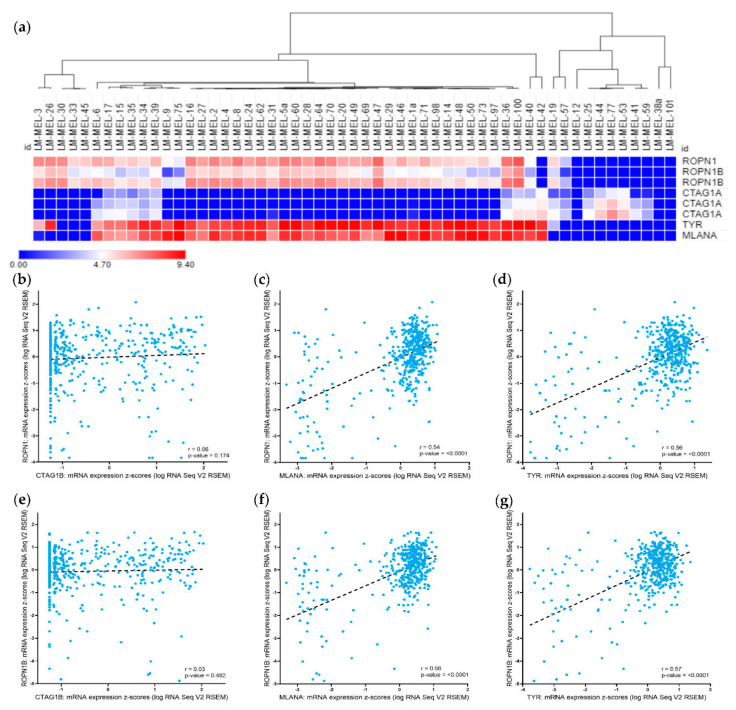
Gene expression of *ROPN1, ROPN1B, CTAG1A/B*, *TYR* and *MLANA* in melanoma cell lines and tumours. (**a**) Heat map representing the hierarchical clustering by Pearson correlation of *ROPN1, ROPN1B, CTAG1A, TYR* and *MLANA* gene expression in 55 in-house generated melanoma cell lines derived from 52 patients [32]. Each row shows absolute level of expression results for one specific probe for the respective gene. The full gene expression dataset can be found under GEO dataset ID GSE89438. (**b**) mRNA co-expression plots for *ROPN1* and *CTAG1B* (*r =* −0.06, *p*-value = 0.174), or (**c**) *TYR* (*r =* 0.56, *p*-value < 0.0001) or (**d**) *MLANA* (*r =* 0.54, *p*-value < 0.0001); and (**e**) mRNA co-expression plots for *ROPN1B* and *CTAG1B* (*r* = 0.03, *p*-value = 0.482), or (**f**) *TYR* (*r =* 0.57, *p*-value < 0.0001) or (**g**) *MLANA* (*r =* 0.56, *p*-value < 0.0001) in 472 TCGA melanoma patient samples (469 patients). mRNA expression is shown as z-scores relative to all samples (log RNA Seq V2 RSEM), and correlation is analysed using the Pearson correlation. Gene Expression Omnibus, GEO; TCGA, The Cancer Genome Atlas.

We tested the gene expression levels of *ROPN1* and *ROPN1B* (96% sequence homology with *ROPN1)* in a panel of melanoma cell lines generated in-house [32] (Table 1). In 45 out of 55 (81.8%) cell lines, we detected *ROPN1* gene expression compared to 46 out of 55 (83.6%) with *ROPN1B* expression (Figure 1A). For the purposes of comparison with a well-validated CTAg, we also determined the gene expression of *CTAG1A* (identical gene copy to *CTAG1B*) in the same cohort of cell lines. *CTAG1A* expression was detected in 20 out of 55 (36.4%) cell lines (Figure 1A), with 14 cell lines showing expression of both CTAgs. Expression of either *ROPN1/ROPN1B* or *CTAG1A* was observed in 52 out of 55 cell lines (94.5%).

The expression of differentiation antigens that are commonly present and have been previously used as immunological targets in melanoma, namely, *TYR* (tyrosinase) and *MLANA* (Melan-A/MART1) [20,33], were compared with *CTAG1A* and *ROPN1* or *ROPN1B* in our panel of melanoma cell lines. We found a strong correlation in the expression of both *TYR* and *MLANA* with *ROPN1* and *ROPN1B* among our cell line panel (Figure 1A, *TYR* vs. *ROPN1*: *r* = 0.73, *p*-value < 0.0001; *TYR* vs. *ROPN1B*: *r* = 0.73, *p*-value < 0.0001; *MLANA* vs. *ROPN1*: *r* = 0.61, *p*-value < 0.0001; *MLANA* vs. *ROPN1B*: *r* = 0.61, *p*-value < 0.0001). In contrast, *CTAG1A* expression was found in more differentiated cell lines with high *TYR* and/or *MLANA* expression, and in less differentiated ones without *TYR* and/or *MLANA* expression (Figure 1A).

We further evaluated the gene expression levels of *ROPN1, ROPN1B, CTAG1B* (identical gene copy to *CTAG1A* for which no gene expression data is available)*, TYR* and *MLANA* in 472 additional melanoma samples using data accessible via The Cancer Genome Atlas (TCGA) (Table 1). As expected, expression of *ROPN1* and its paralog *ROPN1B* was highly correlated (*r* = 0.86, *p*-value = 8.71 × 10^−141^). *ROPN1* and *ROPN1B* were expressed at transcript level in nearly all melanomas (*ROPN1*: *n* = 467/472, 98.9%, *ROPN1B*: *n* = 466/472, 98.7%), more frequently than *CTAG1B* (*n* = 322/472, 68.2%), and sometimes exclusively (Figure 1B, *ROPN1*: *r* = 0.06, *p*-value=0.174; Figure 1E, *ROPN1B*: *r* = 0.03, *p*-value = 0.482). As expected, *TYR* (*n* = 469/472, 99.4%) and *MLANA* (*n* = 471/472, 99.8%) were commonly expressed. We further demonstrated co-expression of *ROPN1*/*ROPN1B* and *TYR* (*ROPN1*: *r* = 0.56, *p*-value < 0.0001; *ROPN1B*: *r* = 0.57, *p*-value < 0.0001) or *MLANA* (*ROPN1*: *r* = 0.54, *p*-value < 0.0001; *ROPN1B*: *r* = 0.56, *p*-value < 0.0001) in the majority of patient samples (Figure 1C, 1F and 1D, 1G, respectively), and found no correlation between differentiation antigens and *CTAG1B* expression (Figure S1, *TYR*: *r* = −0.06, *p*-value=0.165, *MLANA*: *r* = −0.05, *p*-value = 0.268), reflecting the results of our cell line analysis.

We further investigated *ROPN1, ROPN1B* and *CTAG1B* gene expression across different stages of disease and genders in melanoma. *ROPN1*, *ROPN1B* and *CTAG1B* were expressed across all AJCC disease stages, without apparent differences (Figure S2, *ROPN1*: *p*-value = 0.179; *ROPN1B*: *p*-value = 0.140; *CTAG1B*: *p*-value = 0.338). Similarly, no difference was observed between males and females (Figure S3, *ROPN1*: *p*-value = 0.944; *ROPN1B*: *p*-value = 0.362; *CTAG1B*: *p*-value = 0.829).

### 2.2. Ropporin-1B (ROPN1B) Protein Is Expressed in Melanoma Tumours

**Figure 2:**
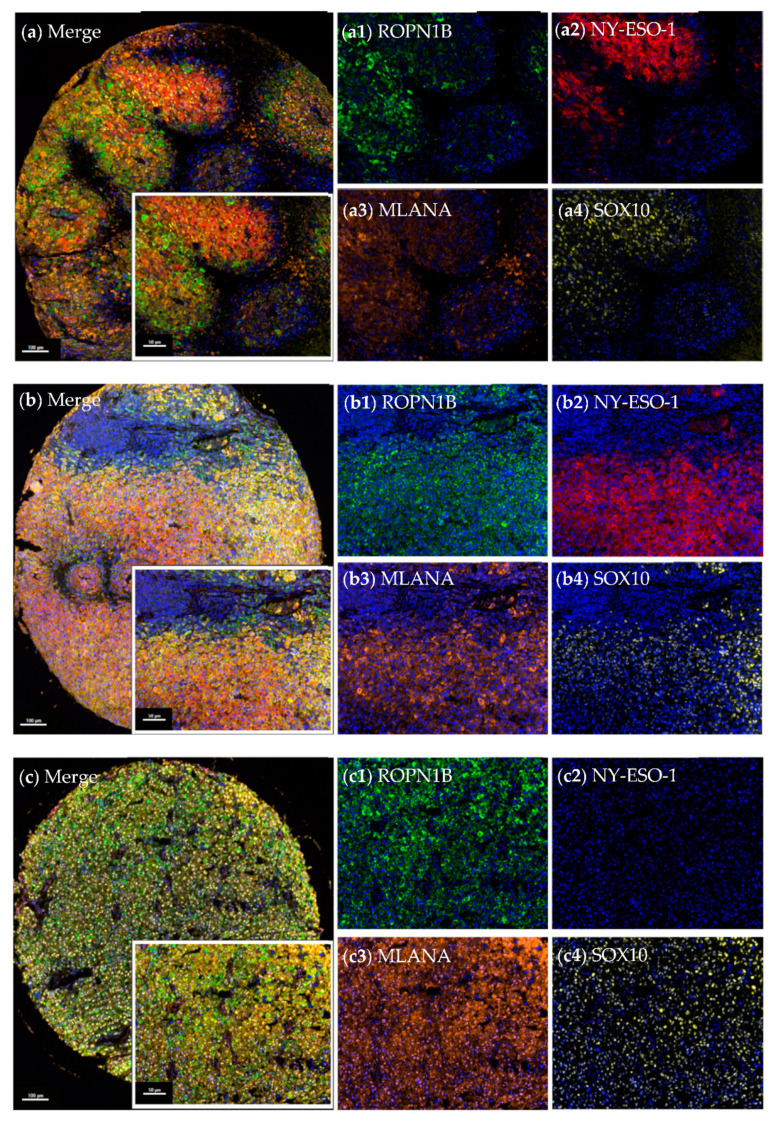
Patterns of ROPN1B and NY-ESO-1 expression in melanoma tumours. Multispectral immunohistochemistry of melanoma tumour cores showing staining for ROPN1B, NY-ESO-1, MLANA and SOX10. Representative cores showing ROPN1B and NY-ESO-1 co-expression in discreet (**a**) or shared (**b**) tumour regions, as well as exclusive (**c**) ROPN1B expression. Tissue microarrays were stained with anti-ROPN1B (green), anti-NY-ESO-1 (red), anti-MLANA (orange) and anti-SOX10 (yellow) antibodies with DAPI (blue) counterstain, and are displayed as merge (**a**,**b**,**c**) and single colour (**a1**–**a4**,**b1**–**b4**, **c1**–**c4**) cores. Whole tumour cores with more than 2% of the tissue cells staining for the target proteins were considered positive. All images were taken using a 20× objective, and scale bars indicate 100 µm (core) or 50 µm (region of interest).

**Figure 3:**
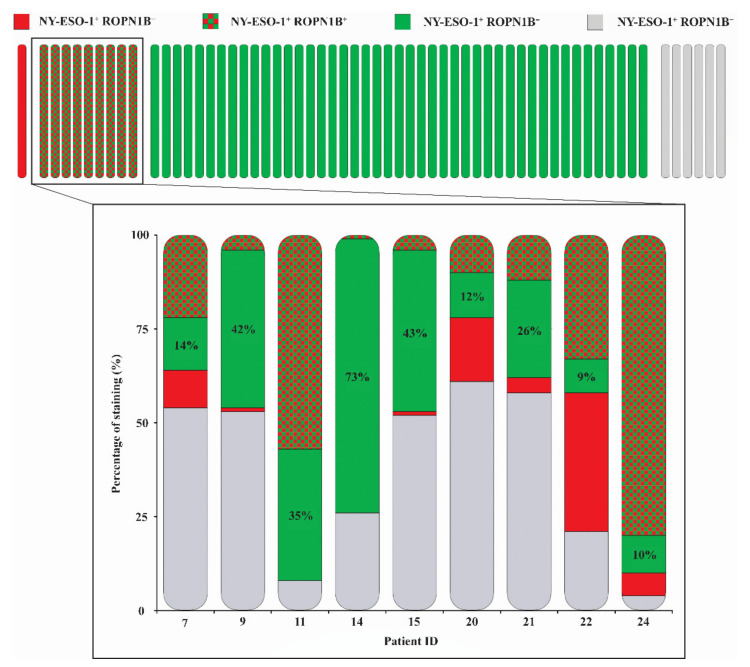
Tumour cell coverage of ROPN1B and NY-ESO-1 expression in investigated melanoma tumours. Upper panel indicates number of tumours with any expression of NY-ESO-1 alone (red), NY-ESO-1 and ROPN1B (chequered red and green), ROPN1B alone (green) or neither (grey) across the cohort. Magnified panel depicts quantitative distribution of NY-ESO-1 and ROPN1B staining relative to SOX10+ melanoma cells across patients displaying both NY-ESO-1 and ROPN1B expression. Percentages on bars show ROPN1B expression alone.

We assessed expression of ROPN1B, NY-ESO-1 (single protein from *CTAG1A* or *CTAG1B* genes), MLANA and SOX10 (used here as a melanoma tumour marker) at the protein level by multispectral immunohistochemistry on melanoma tissue microarrays (TMAs) comprising two or three cores from 61 patient tumours (Table 1). Protein expression levels were at times heterogeneous amongst patient cores, and lack of expression was only reported in cases where all cores per patient were assessable. ROPN1B cytoplasmic expression was observed in 88.5% (*n* = 54/61) of patient samples, while cytoplasmic NY-ESO-1 was observed in 16.4% (*n* = 10/61) of samples (Figure S4). Expression of both ROPN1B and NY-ESO-1 was seen in 9 cases (*n* = 9/61, 14.8%), where tumour cells within cores showed instances of co-expression or exclusive expression of ROPN1B and/or NY-ESO-1 (Figure 2 and Figure 3). Moreover, ROPN1B expression was often detected ubiquitously, whereas NY-ESO-1 appeared more scattered and diffuse throughout tumour cores (Figure S5). In patient samples with expression of both ROPN1B and NY-ESO-1, cells with exclusive ROPN1B positivity increased tumour cell coverage by 29.3% on average (range from 9.0% to 73.0%, Figure 3). Nuclear SOX10 (*n* = 60/61) or cytoplasmic MLANA (*n* = 60/61) expression was detected in nearly all tumour cores, as expected (Figure 2 and Figure S5).

ROPN1B expression was seen in both early (I and II, *n* = 19/26, 73.1%) and late stages (III and IV, *n* = 29/29, 100.0%) of disease, more so than NY-ESO-1 (*n* = 1/26, 3.8% vs. *n* = 9/29, 31.0%, respectively). Nonetheless, expression was more predominant in advanced disease (ROPN1B: chi-square *p*-value = 0.003; NY-ESO-1: chi-square *p*-value = 0.009), with evidence of more abundant melanoma cell expression in tumour cores, in contrast to sparse, dispersed cells commonly identified in early-stage disease. ROPN1B (females: 88.5%, *n* = 23/26 vs. males: 88.6%, *n* = 31/35, chi-square *p*-value = 0.989) and NY-ESO-1 (females: 11.5%, *n* = 3/26 vs. males: 20.0%, *n* = 7/35, chi-square *p*-value = 0.377) protein expression did not differ between genders.

### 2.3. Ropporin-1A (ROPN1A) and Ropporin-1B (ROPN1B) Are Immunogenic Antigens in Melanoma

**Figure 4:**
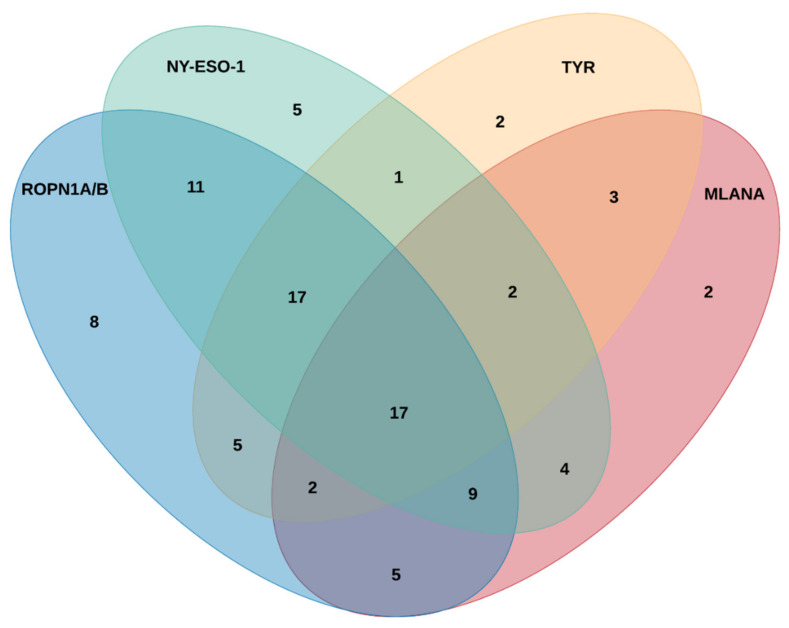
Venn diagram displaying predominance of ROPN1A/B, NY-ESO-1, TYR and MLANA-specific antibodies in melanoma patients. Antibody titres were measured in 104 melanoma patients using a custom protein microarray platform, and all resulting intensities above 500 RFU (defined noise threshold) were considered positive signals and plotted using a 4-way Venn diagram. RFU, relative fluorescent units.

To explore the immunogenic potential of ROPN1A and ROPN1B, we screened sera or plasma from 104 melanoma patients (Table 1). These were tested for the presence and titre of antibodies against ROPN1A/B, NY-ESO-1, MLANA and TYR (Table S2). Antibodies were detected above noise threshold against ROPN1A/B in 71.2% (*n* = 74/104) of patients, and against NY-ESO-1 in 63.5% (*n* = 66/104) of patients. Although there were instances where reactivity was exclusive to either ROPN1A/B (19.2%, *n* = 20/104) or NY-ESO-1 (11.6%, *n* = 12/104), co-reactivity was seen in many cases (51.9%, *n* = 54/104) (Figure 4). Alternatively, a small subset of patients had reactivity to neither (17.3%, *n* = 18/104). In addition, co-reactivity against both ROPN1A/B and TYR (39.4%, *n* = 41/104) or ROPN1A/B and MLANA (31.7%, *n* = 33/104) was also seen, although to a lesser degree. Only 10.6% (*n* = 11/104) of the patient cohort had no antibody reactivity to any of these antigens.

This cohort consisted predominately of stage III and IV melanoma patients, with 74.4% (*n* = 64/86) of these seropositive for ROPN1A/B, and 62.8% (*n* = 54/86) seropositive for NY-ESO-1. Furthermore, it included 60.6% males and 38.8% females, with no gender-related differences seen for ROPN1A/B (females: 75.0%, *n* = 30/40 vs. males: 69.8%, *n* = 44/63, chi-square *p*-value = 0.571) or NY-ESO-1 antibody reactivity (females: 62.5%, *n* = 25/40 vs. males: 63.5%, *n* = 40/63 chi-square *p*-value = 0.919) (Figure S6).

## 3. Discussion

Neoantigens that arise from somatic mutations can play an important role in immune tumour rejection [34] but generally differ from tumour to tumour. In order to create personalised vaccines against these targets, individual therapies need to be tailored on a patient-by-patient basis, requiring gene sequencing and manufacturing of antigens. This approach has limitations for routine clinical use across hospitals both in terms of the associated costs and extensive timeframes. Nevertheless, a small study in melanoma designed personalised neoantigen-based peptide vaccines for six patients, which led to the generation of tumour-specific CD4^+^ and CD8^+^ T lymphocyte responses and clinical benefit (four out of six patients remaining cancer free at a 25-month follow up) [4]. One reason cited for the failure of prior therapies in melanoma, including cancer vaccine- based treatments, has been the considerable heterogeneity of melanoma cells [35,36]. Phenotypic plasticity and differentiation/de-differentiation is a likely contributor to this heterogeneity as it has been known to result in antigen down-regulation. Indeed, epithelial to mesenchymal transition-like phenotype switching has been implicated as an escape mechanism following vaccination or ACT [37,38]. CTAgs comprise a large group of antigens, many of which are expressed following de-repression of epigenetic silencing in cancer [39]. In contrast to the unique products of mutations, their expression is shared between tumours and restricted normal tissues, mostly at immune-privileged sites. Therefore, they are able to stimulate potent immune responses [40] and many have been widely trialled as vaccine antigens [39]. Namely, an NY-ESO-1 vaccine was able to induce antigen-specific cellular and humoral responses but did not affect overall survival, possibly due to its limited tumour expression [41].

In this study, gene-expression profiling of a large panel of early-passage melanoma cell lines [32] identified *ROPN1* and *ROPN1B* as being highly expressed in melanoma tumour samples (*ROPN1*: 81.8%, *ROPN1B*: 83.6%), more so than *CTAG1* (36.4%). Similarly, gene expression of *ROPN1* and *ROPN1B* was also commonly detected in a melanoma patient cohort accessed via the TCGA (*ROPN1*: 98.9%, *ROPN1B*: 98.7% vs. *CTAG1B*: 68.2%). When investigating melanoma TMAs, 88.5% of patient tumours expressed ROPN1B, a much larger proportion compared to NY-ESO-1 (16.4%). Even in instances of tumours expressing both NY-ESO-1 and ROPN1B (14.8%), the addition of ROPN1B increased the tumour cell coverage substantially.

The role of antibodies against tumour antigens in melanoma is unclear [42,43,44], and while an antibody response is not necessarily accompanied by cellular immunity, these often go hand-in-hand [45,46]. Antibody profiling using a custom protein array [47] detected high-titre antibodies against ROPN1A/B in the serum or plasma of a large cohort of melanoma patients (71.2%), more so when compared to NY-ESO-1 (63.5%). NY-ESO-1 antibodies were more commonly detected than expected, based on the above gene and protein expression studies. In previous studies that assessed the frequency of NY-ESO-1 antibody responses, reports have described NY-ESO-1-specific antibodies in ~10% [15] to 45% [48] of patients. However, the protein array used here is more sensitive than ELISA [47], which would contribute to a higher percentage of patients with detectable anti-NY-ESO-1 and/or anti-ROPN1A/B antibody responses when compared to historical data. Alternatively, NY-ESO-1 is known to be highly immunogenic, and hence it is possible that the detected circulating antibodies may be a result of earlier NY-ESO-1^+^ cell eradication. Similarly, the prevalence of detectable antibodies against MLANA and TYR were below expected levels, particularly when considering the above gene expression data, arguing for inferior humoral immunogenicity when compared to ROPN1A/B and NY-ESO-1. Furthermore, a trend towards increasing ROPN1A/B and NY-ESO-1 antibody reactivity was seen with progression of disease stage. For example, 54.5% of Stage I/II had antibody responses to ROPN1A/B, compared with 74.4% of stage III/IV patients. The number of stage I and II patients screened here (*n* = 11) was too small to allow us to draw definitive conclusions, however, prior studies have also demonstrated a correlative increase in the proportion of NY-ESO-1 seropositivity with disease stage [17,49]. In addition, we observed that ROPN1A/B reactivity was slightly increased in females, albeit not significantly. This observation was also noted in a recent study where antibody responses to ROPN1 were significantly higher in female multiple myeloma patients [50]. An increased humoral response in females may explain the reduced melanoma incidence and the increased survival benefit independent of disease stage reported in women [51,52]. Together, these data show that (i) NY-ESO-1, ROPN1 and ROPN1B are highly immunogenic; and (ii) patients who have no evidence of NY-ESO-1 immunity can have antibody titres against other CTAgs, including ROPN1A and ROPN1B, thereby validating that the addition of ROPN1A and ROPN1B as target antigens can potentially enlarge the population of eligible patients who might benefit from a combined vaccine approach.

Ongoing immune editing occurring over the course of disease allows cancer escape from immune targeting of individual antigens [53]. For these reasons, the potential to achieve increased tumour coverage by targeting NY-ESO-1, ROPN1A and ROPN1B combined makes these immunogenic CTAgs highly compatible for combination in a cancer vaccine with the potential to generate synergistic immune responses to the tumour. It is tempting to speculate that further immunogenic tumour antigens could also be incorporated, leading to the design of a cancer vaccine that “covers all bases” in terms of heterogeneity and plasticity. As strategies are being pursued to widen the applicability of cancer immunotherapy, vaccination is now being proposed and trialled (NCT03092453) in combination with other immunotherapies, such as ICB [22,54,55]. Our study indicates that ROPN1A and ROPN1B may serve as promising candidates for such antigen-specific approaches, and therefore further studies are warranted and should be pursued to explore this.

## 4. Materials and Methods

### 4.1. Human Ethics Approval

Blood samples used in this study were derived from patients who provided written informed consent to participate in a clinical research protocol approved by the Austin Health Human Research Ethics Committee (HREC/14/Austin/425, approved on 6 November 2014) (Table 1).

### 4.2. Melanoma Cell Lines and Cell Culture

Establishment and characterisation of the melanoma cell lines used has been previously described [32]. Cells were cultured in RPMI 1640, 2 mM Glutamax, 100 U/mL Penicillin, 100 µg/mL Streptomycin and 10% foetal calf serum (RF10) (all Invitrogen, Carlsbad, CA, USA).

### 4.3. Cell Pellet DNA Extraction

DNA was extracted from pelleted cells using a DNeasy^®^ Blood and Tissue kit (Qiagen, Hilden, Germany) as per the manufacturer’s instructions. Briefly, pellets were suspended in 200 µL of PBS with 36 µL of Proteinase K (600 mAU/mL, Scimar, Templestowe, VIC, Australia) and 200µL AL buffer (Qiagen, Hilden, Germany), then incubated at 56 °C overnight. Clean-up was as per the manufacturer’s instructions and samples were eluted in 50 µL of AE buffer (Qiagen, Hilden, Germany).

### 4.4. Gene Expression—Cell Lines

The gene expression array method and analysis have been previously described (Gene Expression Omnibus, GEO dataset ID GSE89438) [32,56]. Briefly, genomic DNA was purified from 55 melanoma cell lines originating from 52 patients (Qiagen AllPrep kit, Hilden, Germany) and assayed using Illumina standard protocols. Samples were subjected to whole-genome expression arrays (Illumina HT12, San Diego, CA, USA), and hierarchical clustering by Pearson correlation of *ROPN1* (probe ID: 5420739), *ROPN1B* (probe IDs: 730521 and 460291; 96% sequence homology with *ROPN1*), *CTAG1A* (probe IDs: 6770332, 1430215 and 1070577; identical gene copy to *CTAG1B*), *TYR* (probe ID: 5260253) and *MLANA* (probe ID: 7330367) was performed using the Morpheus software (https://software.broadinstitute.org/morpheus/index.html, Broad Institute, Cambridge, MA, USA). Absolute values were used to determine level of expression across cell lines, with any level above 0.3 considered “positive” for expression.

### 4.5. Gene Expression—The Cancer Genome Atlas (TCGA)

The gene expression data of *ROPN1*, *ROPN1B*, *CTAG1B* (identical gene copy to *CTAG1A* for which no gene expression data is available), *TYR* and *MLANA* in melanoma tumour samples was accessible via TCGA research network (http://cancergenome.nih.gov/) and analysed using the cBioPortal [57,58]. The TCGA dataset used was the Skin Cutaneous Melanoma, TCGA, Firehose Legacy, consisting of 472 samples (469 patients). Absolute mRNA transcript values were used to determine level of expression across TCGA patient samples, with any transcript level above zero considered “positive” for expression. mRNA expression of the respective genes is shown as z-scores (RNA Seq V2 RSEM) and represented in a log2 scale. Co-expression of mRNA is analysed using the Pearson correlation.

### 4.6. Multispectral Immunohistochemistry

Two (ME1002b, US Biomax, Derwood, MD, USA and 07 TMA Mel 1.8, in-house) melanoma TMAs containing two or three cores for 61 melanoma patient tumours (59 patients) were baked at 65 °C for 2 h, dewaxed in xylene three times for 10 min, rehydrated in ethanol twice for 10 min and manually stained. The staining included initial blocking of endogenous peroxidases using 3% hydrogen peroxide for 30 min, followed by sequential 15 min rounds of heat-induced epitope retrieval (microwave at 20% power); 10 min blocking of non-specific binding sites; 30 min primary (anti-NY-ESO-1 (single protein from *CTAG1A* or *CTAG1B* genes); ROPN1B, MLANA or SOX10); 10 min secondary (anti-mouse and anti-rabbit Opal™ horseradish peroxidase) antibody incubation; and 10 min fluorophore-tyramide signal amplification using Opal^TM^ 520, 570, 620 and 690 fluorophores labelling target proteins, respectively (Akoya Biosciences^®^, Marlborough, MA, USA) (Table S1). Slides were counterstained with spectral DAPI, and tissue cores were scanned using the Vectra 3 Automated Quantitative Pathology Imaging System (Akoya Biosciences^®^, Marlborough, MA, USA) where target proteins were detected and imaged using the FITC, Cy3, Texas red and Cy5 filter cubes. Images were spectrally unmixed and analysed using inForm^®^ Cell Analysis software version 3.0.5 or HALO™ Image Analysis Software version 3.2 (Akoya Biosciences^®^, Marlborough, MA, USA) with the assistance of a pathologist. Whole tumour cores with more than 2% of the tissue cells staining for the target proteins in at least one out of two or three cores per patient tumour were considered positive. Staining pattern of localization was defined as nuclear (if co-localised with DAPI) or cytoplasmic. Cytoplasmic staining was distinguished from membranous staining by performing immunohistochemistry using anti-MHC class I antibody (membranous expression on melanoma cells that have not experienced MHC class I loss, as well as stromal cells) with either anti-ROPN1B, anti-NY-ESO-1 or anti-MLANA antibodies, along with DAPI counterstain in melanoma tumours.

### 4.7. Antibody Profiling

Serum or plasma from melanoma patients was used to measure antibody titres towards ROPN1A/B, NY-ESO-1, MLANA and TYR using a custom tumour antigen protein microarray platform [47]. Blood samples originated from melanoma patients without available matched tumour tissue. Following the printing of previously expressed biotinylated antigens to a streptavidin-coated microarray slide using a QArray2 robotic arrayer (Genetix, Berkshire, UK), slides were immersed in blocking buffer (50 µM biotin in PBST) and incubated on ice in a plastic chamber protected from light for 1 h. Slides were then washed three times for 5 min in PBST and dried. Individual arrays were incubated with a unique serum or plasma sample (100 µL at 1:800 or 1:400 dilution, respectively) for 1 h at RT, washed in PBST and dH_2_O, and incubated with 100 µL of 20 µg/mL Alexa Fluor 647 Goat anti-Human IgG (H + L) (Invitrogen, Carlsbad, CA, USA, 1:100 dilution in PBST) for 30 min at RT. The individual arrays were then washed, dried and scanned using a Tecan LS Reloaded fluorescence microarray scanner (Tecan Group Ltd., Männedorf, Switzerland) in automatic gain control (AGC) mode. All liquid handling steps were performed using QuadChambers on a Tecan HS4800 Pro automated hybridization station (Tecan Group Ltd., Männedorf, Switzerland). The resulting arrays were viewed using the ArrayPro Analyzer software Version 6.3 (Media Cybernetics, Rockville, MD, USA), and raw data was extracted. Finally, these data were processed using a custom bioinformatic tool for protein microarray data processing and normalisation, and the resulting data were analysed accordingly [59].

## 5. Conclusions

In this study, we have identified ROPN1 and ROPN1B as compelling novel therapeutic antigen targets for further clinical evaluation. Features include frequent humoral immunogenicity and abundance, with virtually universal tumour-specific expression across all melanomas tested.

## Figures and Tables

**Table 1:** Summary of patient characteristics across all cohorts. This includes sample numbers, age, gender and disease stage. Cohort 1 was used to generate gene expression data (GEO dataset ID GSE89438) from 55 in-house generated melanoma cell lines derived from 52 patients [32]. Cohort 2 was used to generate gene expression data from 472 melanoma patient tumours derived from the TCGA Skin Cutaneous Melanoma Firehose Legacy dataset with 469 patients. Cohort 3 was used to generate protein expression data from 61 melanoma patient tumours across 2 TMAs (ME1002b, US Biomax, Derwood, MD, USA and 07 TMA Mel 1.8, in-house) derived from 61 patients. Cohort 4 was used to generate circulating antibody data from 104 melanoma patient serum or plasma. Gene Expression Omnibus, GEO; TCGA, The Cancer Genome Atlas; TMAs, tissue microarrays; yr, years.

	Cohort 1 (*n* = 52)	Cohort 2 (*n* = 469)	Cohort 3 (*n* = 61)	Cohort 4 (*n* = 104)
Sample Type	Cell lines	Tumours	Tumours	Serum or Plasma
Used to Determine	Gene Expression	Gene Expression	Protein Expression	Circulating Antibodies
Age–yr				
Median	56	58	53	54
Range	25–83	15–90	21–88	21–87
Gender–no. (%)				
Unknown	0 (0)	0 (0)	0 (0)	1 (0.9)
Male	33 (63.5)	289 (61.6)	35 (57.4)	63 (60.6)
Female	19 (36.5)	180 (38.4)	26 (42.6)	40 (38.5)
Stage–no. (%)				
Unknown	9 (17.3)	59 (12.6)	6 (9.8)	7 (6.7)
I	0 (0)	77 (16.4)	5 (8.2)	2 (1.9)
II	1 (1.9)	140 (29.9)	21 (34.4)	9 (8.7)
III	22 (42.3)	170 (36.2)	4 (6.6)	35 (33.7)
IV	20 (38.5)	23 (4.9)	25 (41.0)	51 (49.0)

## Data Availability

Publicly available datasets were analysed in this study. This data can be found here: https://www.ncbi.nlm.nih.gov/geo/query/acc.cgi?acc=GSE89438; and here: http://www.cbioportal.org/study/summary?id=skcm_tcga. The remaining data presented in this study are available within the article or in the supplementary material.

## Supplementary Materials

The following are available online at https://www.mdpi.com/article/10.3390/cancers13081805/s1, Figure S1: Gene expression of *CTAG1B* and differentiation antigens *TYR* and *MLANA* in melanoma tumours, Figure S2: Gene expression of *ROPN1, ROPN1B* and *CTAG1B* by AJCC disease stage codes in melanoma tumours, Figure S3: Gene expression of *ROPN1, ROPN1B* and *CTAG1B* by gender in melanoma tumours, Figure S4: Cytoplasmic expression of ROPN1B, NY-ESO-1 and MLANA in melanoma tumours, Figure S5: Variability seen between concentrated ROPN1B and diffuse NY-ESO-1 staining patterns in melanoma tumours, Figure S6: Venn diagram displaying predominance of ROPN1A/B and NY-ESO-1-specific antibodies in melanoma patients by gender, Table S1: Specifications of antibodies and conditions used for multispectral immunohistochemistry, Table S2: ROPN1A, ROPN1B, NY-ESO-1, MLANA and TYR-specific antibodies in melanoma patients.
