# Supplementary material for: Ropporin-1 and 1B Are Widely Expressed in Human Melanoma and Evoke Strong Humoral Immune Responses
Source: Cancers (Basel). 2021 Apr 9;13(8):1805. doi: 10.3390/cancers13081805 (PMC8069442; doi:10.3390/cancers13081805)
Supplement: Supplementary file 1 [file cancers-13-01805-s001.pdf]

SUPPLEMENTARY FIGURES

Figure S1. Gene expression of *CTAG1B* and differentiation antigens *TYR* and *MLANA* in melanoma tumours. (**a**) mRNA co-expression of *CTAG1B* and *TYR* (r=-0.06, p-value=0.165) or **(b)** *MLANA* (r=-0.05, p-value=0.268) in melanoma patients using the TCGA dataset (Skin Cutaneous melanoma, TCGA, Firehose Legacy) consisting of 472 samples (469 patients). mRNA expression is shown as z-scores relative to all samples (log RNA Seq V2 RSEM), and correlation is analysed using the Pearson correlation. TCGA, The Cancer Genome Atlas.

(**a**)

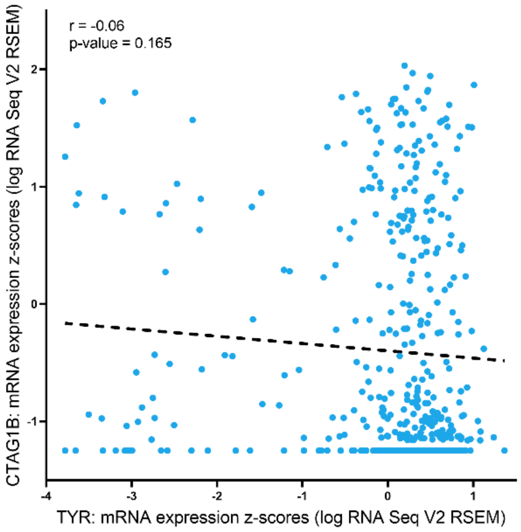

(**b**)

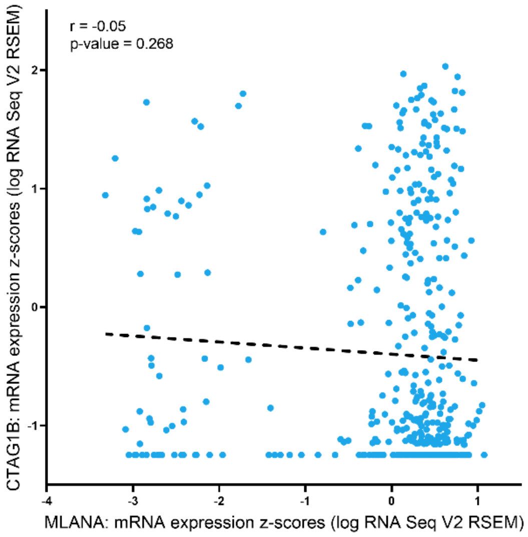

Figure S2. Gene expression of *ROPN1*, *ROPN1B* and *CTAG1B* by AJCC disease stage codes in melanoma tumours. (**a**) mRNA expression of *ROPN1* (p-value=0.1793), (**b**) *ROPN1B* (p-value=0.1396) and **(c)** *CTAG1B* (p-value=0.3375) in melanoma patients according to the Neoplasm Disease Stage AJCC codes using the TCGA dataset (Skin Cutaneous melanoma, TCGA, Firehose Legacy) consisting of 472 samples (469 patients). mRNA expression is shown as z-scores relative to all samples (log RNA Seq V2 RSEM). TCGA, The Cancer Genome Atlas.

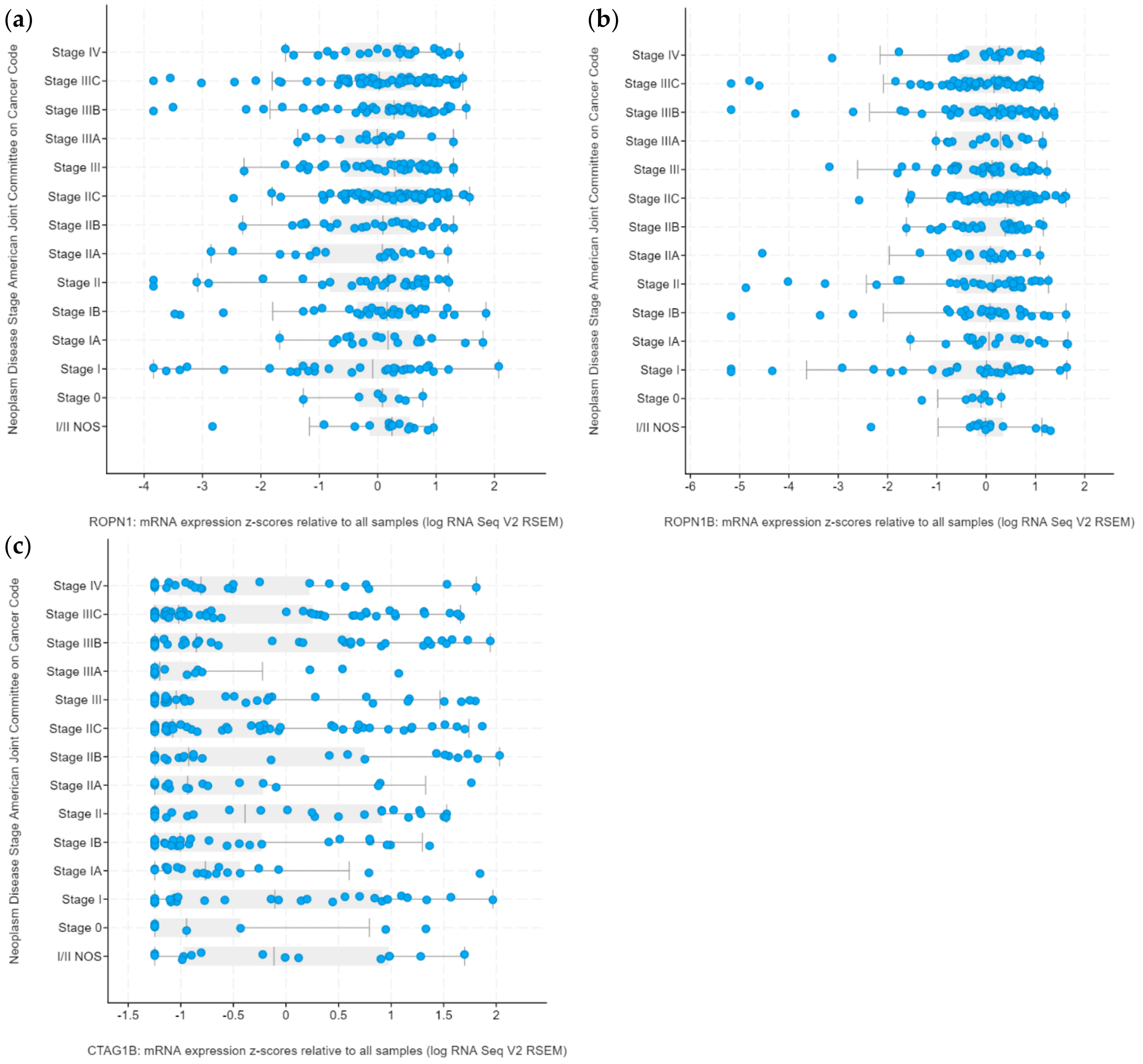

Figure S3. Gene expression of *ROPN1, ROPN1B* and *CTAG1B* by gender in melanoma tumours. (**a**) mRNA expression of *ROPN1* (p-value= 0.9437), (**b**) *ROPN1B* (p-value=0.3623) and (**c**) *CTAG1B* (p-value=0.8291) in melanoma patients according to gender using the TCGA dataset (Skin Cutaneous melanoma, TCGA, Firehose Legacy) consisting of 472 samples (469 patients). mRNA expression is shown as z-scores relative to all samples (log RNA Seq V2 RSEM). TCGA, The Cancer Genome Atlas.

(**a**)

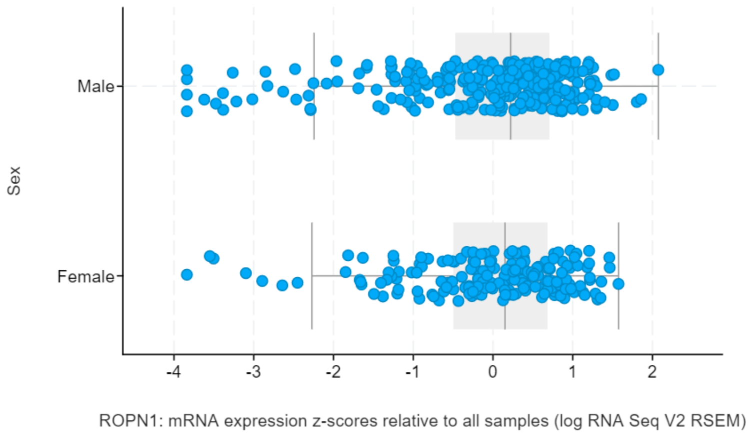

(**b**)

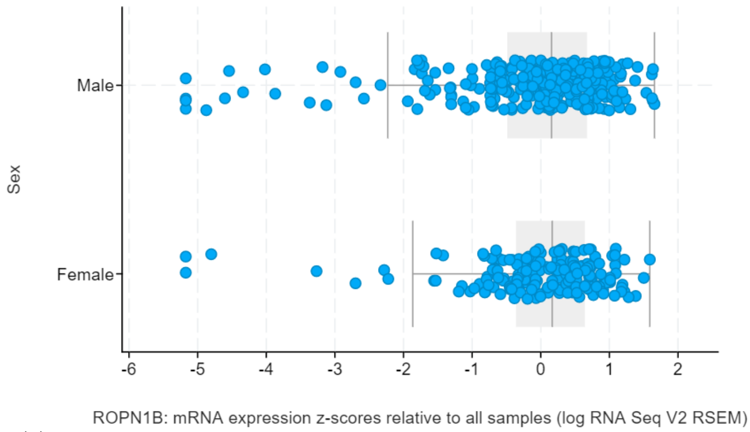

(**c**)

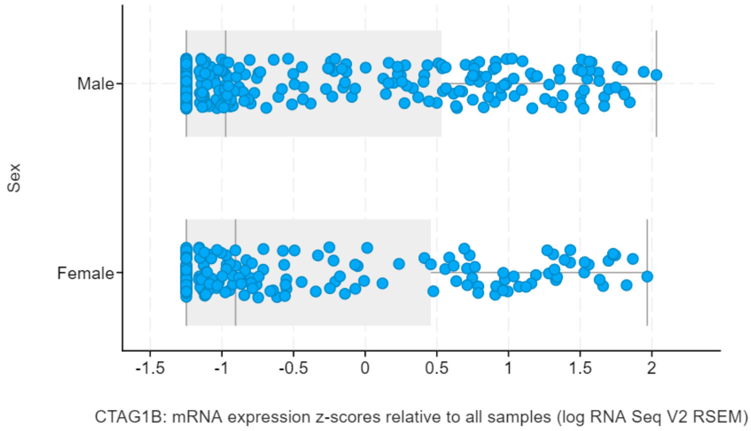

Figure S4. Cytoplasmic expression of ROPN1B, NY-ESO-1 and MLANA in melanoma tumours. Multispectral immunohistochemistry of melanoma tumour cores showing staining for ROPN1B, NY-ESO-1 or MLANA with MHC class I. Representative region of interest showing ROPN1B (**a1**) and MHC Class I (**a2**), NY-ESO-1 (**b1**) and MHC Class I (**b2**), or MLANA (**c1**) and MHC Class I (**c2**) staining patterns. Tissue microarrays were stained with anti-ROPN1B (green), anti-NY-ESO-1 (red) or anti-MLANA (orange) and anti-MHC Class I (white) antibodies with DAPI (blue) counterstain, and are displayed as merge (**a, b and c**) and single colours (**a1, a2, b1, b2, c1 and c2**). All images were taken using a 40x objective, and scale bars indicate 50μm or 100μm.

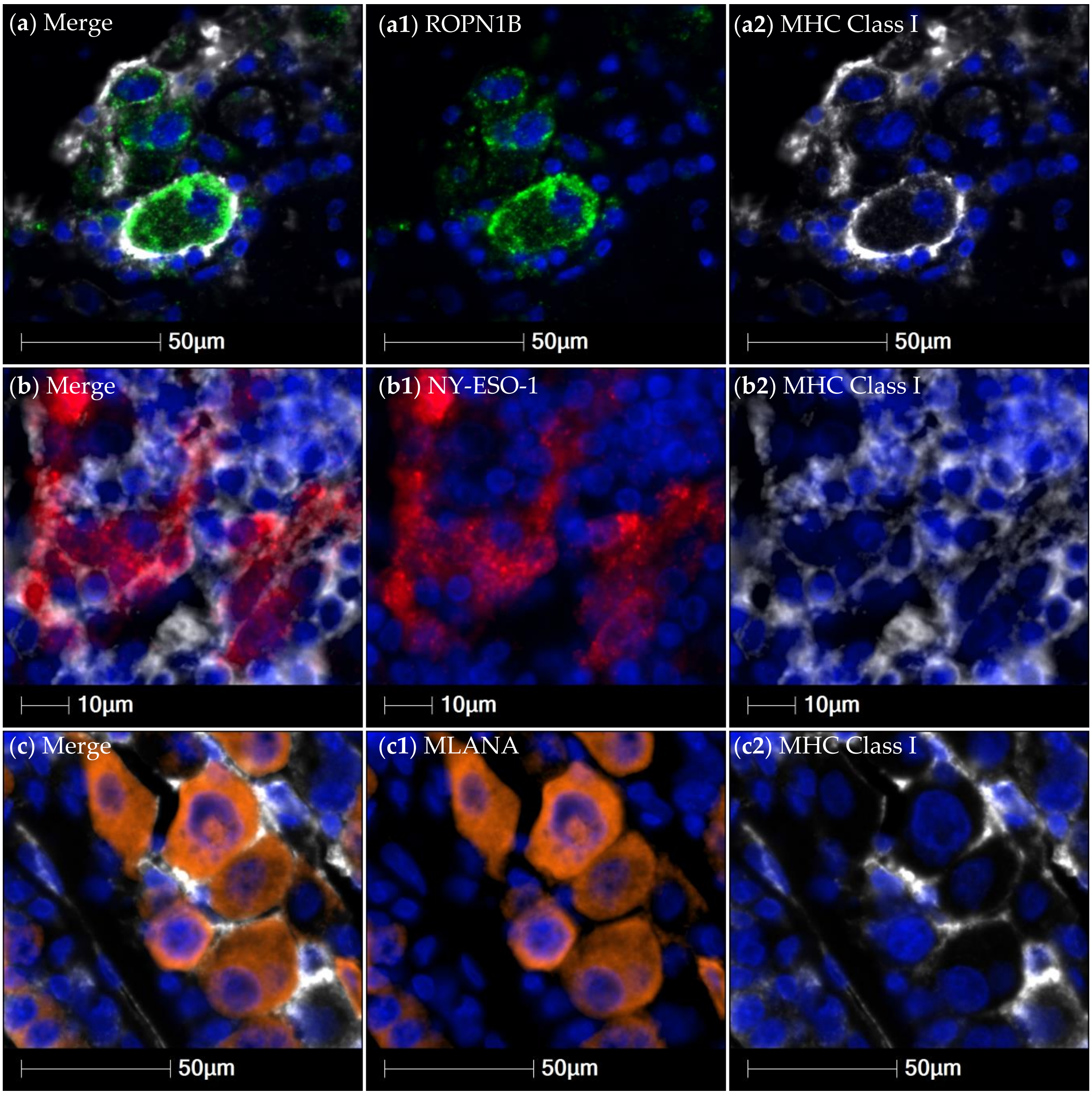

Figure S5. Variability seen between concentrated ROPN1B and diffuse NY-ESO-1 staining patterns in melanoma tumours. Multispectral immunohistochemistry of a melanoma tumour core showing staining for ROPN1B, NY-ESO-1, MLANA and SOX10. (**a**) Representative region of interest showing ROPN1B (**a1**) and NY-ESO-1 (**a2**) staining patterns. Tissue microarrays were stained with anti-ROPN1B (green), anti-NY-ESO-1 (red), anti-MLANA (orange) and anti-SOX10 (yellow) antibodies with DAPI (blue) counterstain, and are displayed as merge (**a**) and single colour (**a1-a4**) cores. Whole tumour cores with more than 2% of the tissue cells staining for the target proteins were considered positive. All images were taken using a 20x objective, and scale bars indicate 100μm (core) or 50μm (region of interest).

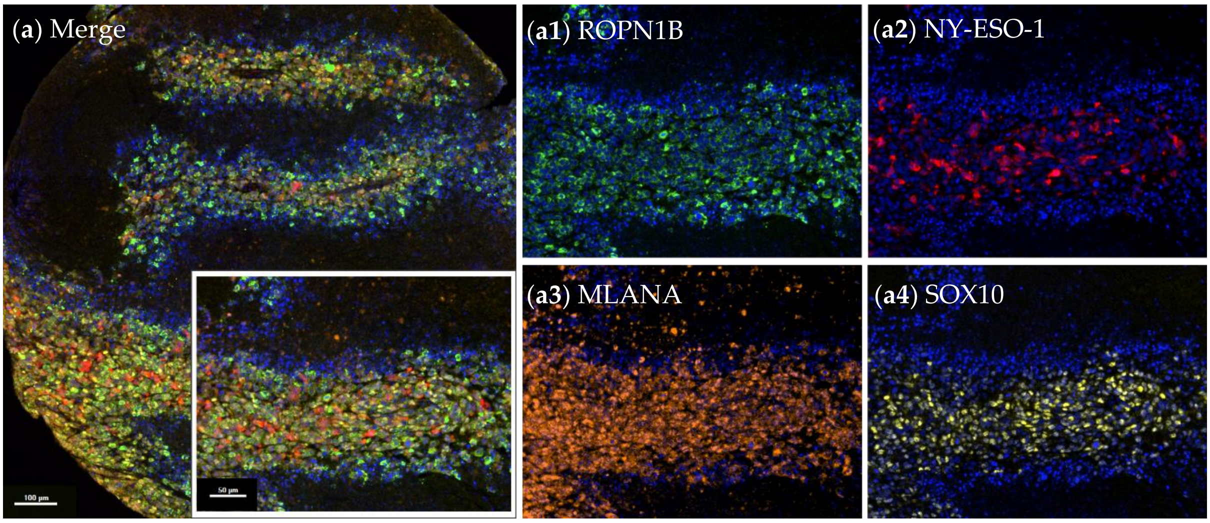

Figure S6. Venn diagram displaying predominance of ROPN1A/B and NY-ESO-1-specific antibodies in melanoma patients by gender. Antibody titres were measured in 104 melanoma patients using a custom protein microarray platform, and all resulting intensities above 500 RFU (defined noise threshold) were considered positive signals and plotted using 2-way Venn diagrams. RFU, relative fluorescent units.

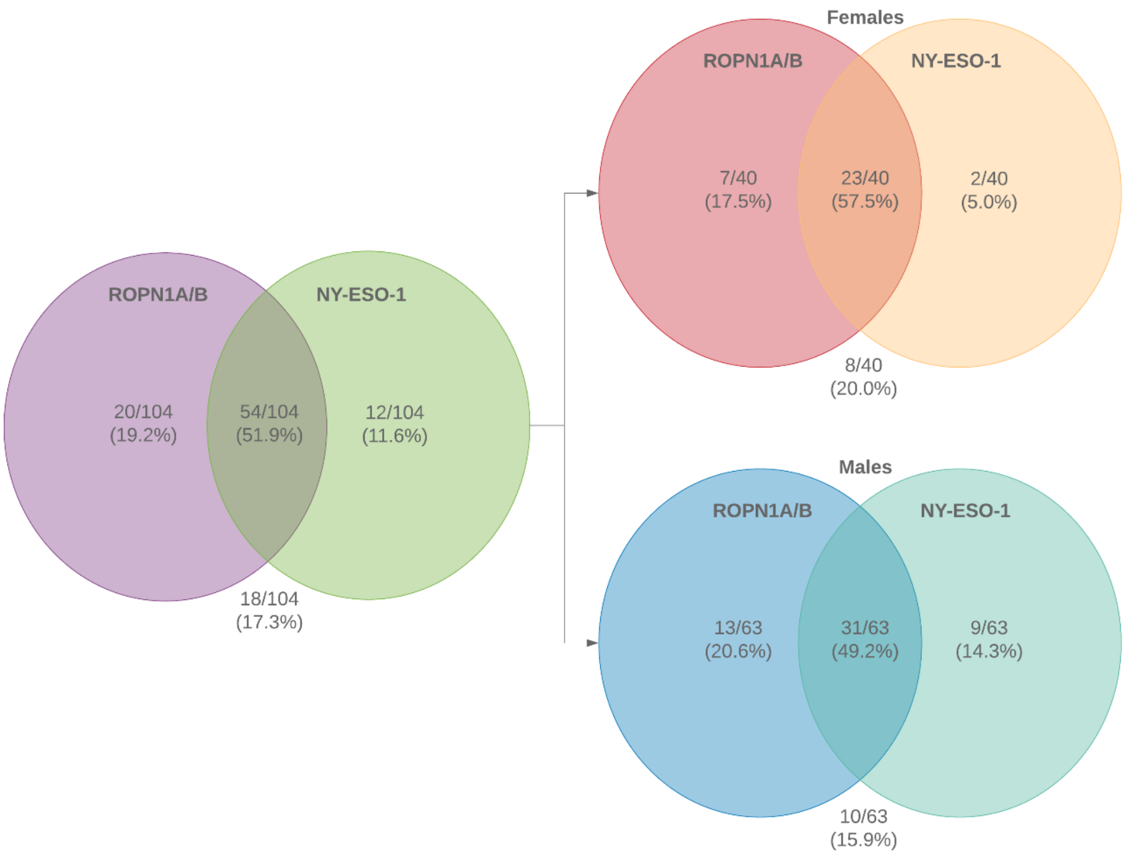

SUPPLEMENTARY TABLES

Table S1. Specifications of antibodies and conditions used for multispectral immunohistochemistry.

Primary monoclonal antibody	Clone	Optimal concentration	Epitope retrieval
ROPN1B (Sigma-Aldrich, St. Louis, MO, USA, # HPA052530)	Polyclonal	1:500 dilution	pH 6
NY-ESO-1 (Invitrogen, Carlsbad, CA, USA, #35-6200)	E978	1:100 dilution, 5μg/mL	pH 9
MLANA (Novus Biologicals, Littleton, CO, USA, #NBP1-30151)	A19-P	1:100 dilution	pH 9
SOX-10 (Abcam, Cambridge, UK, #ab195364)	BC34	1:100 dilution, 125μg/mL	pH 9
MHC Class I (in-house)	-	1:3000 dilution	pH 9

Table S2. ROPN1A, ROPN1B, NY-ESO-1, MLANA and TYR-specific antibodies in melanoma patients. Antibody titres were measured in 104 melanoma patients using a custom protein microarray platform, and all resulting intensities above 500 RFU (defined noise threshold) were considered positive signals.

Patient ID	ROPN1A	ROPN1B	NY-ESO-1	MLANA	TYR
ONJ001	0	0	0	0	681
ONJ002	1925	945	965	1225	1213
ONJ003	0	1367	0	0	2051
ONJ004	1593	2627	121295	1239	1445
ONJ005	1670	0	1635	0	0
ONJ006	0	3198	269600	0	15626
ONJ007	663	695	30877	0	0
ONJ008	0	552	586	0	0
ONJ009	0	1649	2036	0	3081
ONJ010	0	0	796	554	0
ONJ011	0	1769	1785	0	2268
ONJ012	1388	0	1499	0	0
ONJ013	3932	0	6459	0	3732
ONJ014	1387	0	1579	0	0
ONJ015	2338	2081	1566	2449	3790
ONJ016	2334	1747	1608	2215	0
ONJ017	2407	1351	0	2152	0
ONJ018	525	0	1422	0	0
ONJ019	840	603	2416	0	0
ONJ020	890	0	84356	1166	1068
ONJ021	684	902	43316	659	734
ONJ022	0	0	0	656	562
ONJ023	703	592	0	908	0
ONJ024	0	0	0	0	0
ONJ025	735	619	826	997	0
ONJ026	913	675	70682	0	1089
ONJ027	703	0	0	0	996
ONJ028	623	1939	2121	2823	3948
ONJ029	0	506	9087	550	580
ONJ030	0	0	0	0	0
ONJ031	1315	545	24225	616	588
ONJ032	1077	0	74036	1305	1195
ONJ033	980	0	54590	764	1627
ONJ034	868	1380	95646	2526	2570
ONJ035	0	505	27108	0	0
ONJ036	0	0	0	0	0
ONJ037	988	667	18504	1012	1044
ONJ038	0	0	691	959	594
ONJ039	519	513	23685	508	0
ONJ040	592	676	0	543	0
ONJ041	0	846	530	0	806
ONJ042	0	1536	623	0	623
ONJ043	0	0	78710	1213	1002
ONJ044	585	586	0	614	0
ONJ045	687	657	62609	1096	2428
ONJ046	842	0	0	0	0
ONJ047	0	812	15263	0	511
ONJ048	1529	1209	90102	1457	0
ONJ049	695	0	0	0	0
ONJ050	962	529	1248	0	968

Table S2 continued.

Patient ID	ROPN1A	ROPN1B	NY-ESO-1	MLANA	TYR
ONJ051	883	0	1232	0	0
ONJ052	0	672	602	0	511
ONJ053	641	846	0	2130	3056
ONJ054	0	0	0	4476	2304
ONJ055	531	0	14810	0	0
ONJ056	2223	892	1666	503	556
ONJ057	737	717	0	0	589
ONJ058	987	0	2045	0	2254
ONJ059	577	0	637	666	646
ONJ060	652	602	0	0	682
ONJ061	0	0	0	691	0
ONJ062	0	0	578	0	0
ONJ063	0	0	614	0	0
ONJ064	0	0	507	0	0
ONJ065	0	0	2787	1084	0
ONJ066	0	817	0	0	0
ONJ067	0	558	0	0	0
ONJ068	0	7621	26164	0	0
ONJ069	0	0	0	0	0
ONJ070	0	0	0	0	0
ONJ071	935	0	0	0	0
ONJ072	0	552	704	926	0
ONJ073	0	1851	50556	837	1536
ONJ074	0	1015	524	672	0
ONJ075	0	0	0	0	0
ONJ076	0	0	0	0	10613
ONJ077	0	0	502	0	0
ONJ078	969	692	1458	0	817
ONJ079	540	553	512	0	562
ONJ080	0	1879	78437	2010	0
ONJ081	0	0	0	848	0
ONJ082	0	0	88337	1571	0
ONJ083	524	0	2755	0	759
ONJ084	0	0	121254	0	3846
ONJ085	0	0	783	0	0
ONJ086	621	0	0	1324	543
ONJ087	0	515	0	0	0
ONJ088	0	623	0	0	0
ONJ089	0	0	0	0	0
ONJ090	0	0	644	777	0
ONJ091	0	0	0	0	0
ONJ092	1034	0	0	0	0
ONJ093	584	612	580	0	725
ONJ094	0	0	0	0	0
ONJ095	1077	1109	68175	1714	0
ONJ096	872	0	1785	0	596
ONJ097	951	0	0	560	0
ONJ098	0	0	0	859	540
ONJ099	1093	0	1631	0	502
ONJ100	0	0	0	0	0
ONJ101	0	613	0	0	543
ONJ102	508	0	970	851	837
ONJ103	0	0	0	0	0
ONJ104	0	0	2489	602	0
